# *Notes from the Field:* Carbapenemase-Producing *Klebsiella pneumoniae* in a Ventilator-Capable Skilled Nursing Facility — Maricopa County, Arizona, July–November 2018

**DOI:** 10.15585/mmwr.mm6910a5

**Published:** 2020-03-13

**Authors:** Sarah E. Scott, James Matthews, Katherine C. Hobbs, Keila Maldonado, Rachana Bhattarai, Rebecca Sunenshine, Siru Prasai

**Affiliations:** ^1^Epidemic Intelligence Service, CDC; ^2^Maricopa County Department of Public Health, Phoenix, Arizona; ^3^Arizona Department of Health Services; ^4^Career Epidemiology Field Officer Program, Division of State and Local Readiness, Center for Preparedness and Response, CDC.

On August 2, 2018, Maricopa County (Arizona) Department of Public Health (MCDPH) identified two isolates of carbapenemase-producing *Klebsiella pneumoniae* (KPC-KP), a type of carbapenemase-producing carbapenem-resistant Enterobacteriaceae (CP-CRE), from urine specimens collected on July 17 and July 23 from two residents of a ventilator-capable unit in a skilled nursing facility. CP-CRE are multidrug-resistant organisms typically isolated from persons with a health care exposure (*1*,*2*). Invasive CP-CRE infections are associated with a 50% case-fatality rate (*3*); however, only 31%–63% of asymptomatic carriers are identified with clinical cultures (*4*,*5*) and might serve as sources of CP-CRE transmission. Both residents at this skilled nursing facility had indwelling urinary catheters and urinary tract infections, resided in neighboring rooms, and were dependent on nursing care for their activities of daily living; one resident was mechanically ventilated. The Antibiotic Resistance Laboratory Network Mountain Region laboratory in Austin, Texas, performed pulsed-field gel electrophoresis (PFGE) on the two clinical isolates, which were found to have indistinguishable PFGE patterns, suggesting health care–associated transmission. MCDPH and the Arizona Department of Health Services (ADHS) investigated the cluster to prevent additional cases.

MCDPH recommended that the ventilator-capable unit perform contact screening for KPC-KP colonization by rectal swab and culture. The skilled nursing facility had 192 resident beds, 48 (25%) of which were in the ventilator-capable unit; the average length of stay was 14 days. A case was defined as isolation of KPC-KP with a PFGE pattern indistinguishable from that of the two index patients from any specimen source collected from a resident of the ventilator-capable unit during July–November 2018. Contacts were defined as residents residing for ≥3 days in the same ventilator-capable unit as either of the two index patients. On August 13, among 42 identified contacts, six (14%) declined screening, seven (17%) had been discharged, two (5%) were deceased, and one (2%) had a recent infection with a different carbapenem-resistant organism. Among the remaining 26 (62%) residents who were screened, KPC-KP isolates were detected in five (19%) asymptomatic contacts, three of which had indistinguishable PFGE patterns from those of the two index patients.

On September 6, MCDPH and ADHS conducted a site visit to the facility to observe infection control practices with emphasis on the ventilator-capable unit and recommend targeted control measures. Observations included missed opportunities for hand hygiene before and after physical contact with residents and lapses in aseptic technique during routine sterile procedures. MCDPH recommended housing residents with CP-CRE infection in the same ward or in the same room when possible; implementing contact precautions with room restriction for residents with CP-CRE infection who are mechanically ventilated, have tracheostomies, or have uncontained body fluids; requiring staff members to perform hand hygiene with alcohol-based hand sanitizer before and after physical contact with residents; increasing access to alcohol-based hand sanitizer by installing additional dispensers; and offering trainings to staff members for commonly performed sterile procedures.

On November 5, contacts were rescreened to determine whether recommended control measures were successful in containing the cluster. Twenty-eight residents, none of whom had had KPC-KP isolates detected previously, were identified using the previous criteria for rescreening; nine (32%) declined and 19 (68%) consented, 10 of whom had been screened previously. All 19 (100%) rescreened cultures were negative for KPC-KP. Both index patients were treated with antibiotics for KPC-KP urinary infections, and neither died. Following this investigation, one patient had multiple urine specimens collected in which a KPC-KP isolate was identified, suggesting urinary colonization.

Among 26 screened ventilator-capable unit contacts, the investigation identified three (12%) additional cases of KPC-KP colonization with an isolate that had an indistinguishable PFGE pattern from that of the two index patients, which supported health care–associated transmission. Closer adherence to CDC recommendations that could prevent health care–associated KPC-KP transmission include housing together residents with infection, improving adherence to hand hygiene, using gowns and gloves when interacting with residents who require mechanical ventilation or have tracheostomies, and implementing contact precautions for uncontained body fluids (*6*).
